# Is Time Scheduling Important? An Analysis of Donor Heart Cross-clamp Times During Heart Transplantation

**DOI:** 10.1097/TXD.0000000000001588

**Published:** 2024-03-22

**Authors:** Doug A. Gouchoe, Asvin M. Ganapathi, Ervin Y. Cui, Matthew C. Henn, Wai Yen Yim, Bingchuan Geng, Bryan A. Whitson, Hua Zhu

**Affiliations:** 1 Department of Surgery, The Ohio State University Wexner Medical Center, Columbus, OH.; 2 Department of Cardiovascular Surgery, Union Hospital, Tongji Medical College, Huazhong University of Science and Technology, Wuhan, China.; 3 The Davis Heart and Lung Research Institute, The Ohio State University Wexner Medical Center, College of Medicine, Columbus, OH.

## Abstract

**Background.:**

Outcomes in heart transplantation are affected by a variety of variables and patient factors. However, the impact of circadian rhythms, gene expression, and transcription remain underexplored. We thus evaluated the potential role of donor heart cross-clamp times on short-term and long-term outcomes after heart transplantation.

**Methods.:**

A total of 31 713 heart transplants were identified from the United Network for Organ Sharing Database. Patients were first stratified on the basis of time of donor procurement: 12 am to 12 pm or 12 pm to 12 am. To evaluate a possible effect of circadian rhythms, donor time was further divided into 5 groups based on preclinical data: 4 am to 8 am; 8 am to 11 am; 11 am to 5 pm; 5 pm to 10 pm; 10 pm to 4 am. Groups were assessed with comparative statistics. Long-term survival was evaluated using Kaplan-Meier methods and a multivariate Cox proportional hazard model.

**Results.:**

Patients who received hearts recovered between 12 am and 12 pm had significantly higher survival than those who received hearts recovered between 12 pm and 12 am. This survival difference was observed in both unadjusted (*P* = 0.002) and adjusted analyses (hazard ratio [HR]: 0.93; 95% confidence interval [CI], 0.89-0.97; *P* < 0.001). On unadjusted analysis, the survival difference among the 5 groups was insignificant (*P* = 0.07). Following adjustment, the periods of 11 am to 5 pm (HR: 1.09, 95% CI, 1.02-1.17; *P* = 0.012), 5 pm to 10 pm (HR: 1.11; 95% CI, 1.04-1.19; *P* = 0.002), and 10 pm to 4 am (HR: 1.07; 95% CI, 1.01-1.15; *P* = 0.034), were all independently associated with increased long-term mortality. Notably, the time of 8 am to 11 am was not associated with a change in survival (HR: 1.04; 95% CI, 0.96-1.14; *P* = 0.3).

**Conclusions.:**

Given the independent association of donor timing and survival after adjustment in a large national cohort, further investigation into the role of donor circadian rhythm and donor procurement time is warranted in preclinical and clinical studies. Understanding the underlying mechanisms of this observation could potentially lead to the development of effective treatments and donor procurement processes that prepare the organs for transplantation in a better condition.

## Graphical abstract

**Figure:**
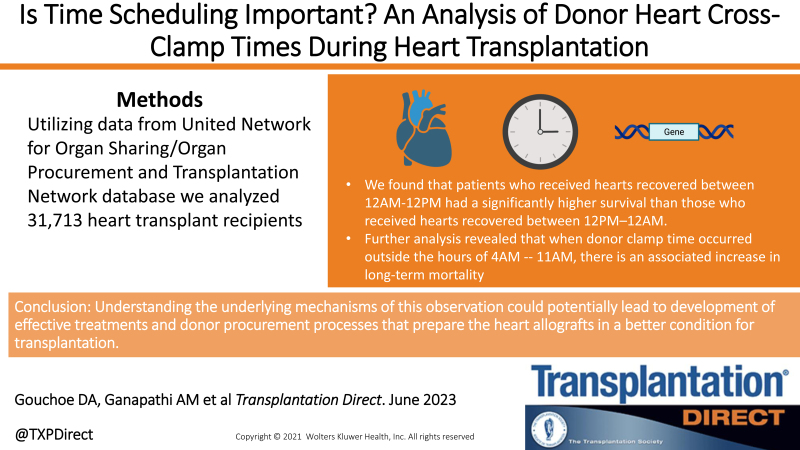

Unlike elective surgical practices, the timing of transplantation surgery is dependent on organ availability and is not adherent to a set schedule. Operative timing is often set through an amalgamation of donor family, procurement team, donor or availability, and recipient or availability. Organ procurements can occur at any time of the day and require complex teamwork, careful coordination, and dedication from all providers. Outcomes in heart transplantation are affected by various variables and patient factors. In the past, researchers have sought to determine whether operative timing played a role in heart transplantation outcomes because irregular hours can have an untold effect on both patient and provider. Large database studies examining both heart and lung transplantation have found there is no significant association between operative timing and short-term mortality.^1^ This has been further substantiated by single-intuitional data when examining heart transplants.^2^ Specifically, with circadian rhythms in mind, Immohr et al^3^ investigated circadian rhythms and cardiac transplantation using single-institutional data found no difference in short or long-term outcomes based on the time of the start of the recipient operation. However, the impact of circadian rhythms, gene expression, and transcription remains underexplored. In this analysis, using the United Network for Organ Sharing (UNOS)/Organ Procurement and Transplant Network Database, we sought to determine whether there is a potential role of donor heart cross-clamp times of heart in affecting the outcomes of heart transplantation.

## MATERIALS AND METHODS

Adult heart and lung transplants were identified using data from the UNOS/Organ Procurement and Transplant Network database from June 1, 2006, to March 31, 2021. Recipients were excluded if they had a prior heart transplant, had multiorgan transplant, or if they had a diagnosis of congenital heart disease. Patients were first stratified on the basis of time of donor procurement: daytime, 12 am to 12 pm (DT) or nighttime, 12 pm to 12 am (NT).^4^ Given this broad range in the initial analysis, donor time was further divided into 5 groups: T1 (4 am–8 am), T2 (8 am–11 am), T3 (11 am–5 pm), T4 (5 pm–10 pm), and T5 (10 pm–4 am) based on prior comprehensive molecular analyses of the circadian transcription cycle^5^ in preclinical studies in a murine model. The study was exempt from institutional review board approval (no. #2018H0079).

### Statistical Analysis

Continuous variables were assessed for normality using QQ plots and presented as mean ± SD (parametric) or median (interquartile range [IQR]) (nonparametric). Missingness was determined in all variables. All groups were then compared using analysis of variance (continuous parametric), the Kruskal-Wallis test (continuous nonparametric), or the chi-square test (categorical).

Unadjusted long-term survival was assessed using Kaplan-Meier methods with the log-rank test. A multivariable Cox proportional hazard model was created to adjust for recipient, donor, and transplant variables. Recipient variables included age, gender, ethnicity, body mass index (BMI), smoking history, diabetes, glomerular filtration rate (GFR), diagnosis before transplant, hospitalization status, preoperative ventilator, preoperative mechanical circulatory support (MCS) including durable left ventricular assist device (LVAD), intra-aortic balloon pump, temporary ventricular assist device (VAD), biventricular assist device (BIVAD), total artificial heart (TAH) and extracorporeal membrane oxygenation (ECMO), and time on the waitlist. Donor variables included age, BMI, diabetes, smoking history, hypertension, and death mechanism.

Transplant-related variables included yearly center volume, distance ischemia time in hours, length of stay (LOS) in days, and era of transplantation (2006–2011, 2012–2017, and 2018–2021). Variables were chosen on the basis of clinical experience.

All statistical analyses were performed with R version 3.6.2 (R Core Team, Vienna, Austria). Statistical significance was set at a *P* value of <0.05 for all analyses.

## RESULTS

After querying the UNOS database, a total of 31 713 heart transplant recipients were identified.

### Results Based on the 2 Category Time Split

Regarding heart transplant recipients, there were 16 455 (51.89%) patients in NT and 15 258 (48.11%) patients in DT. Patients in DT were more often White, had a significantly higher creatinine, and had lower GFR. Preoperative diagnosis and the use of preoperative MCS did not differ significantly among the groups, nor did inotrope use. Additional recipient demographics can be seen in Table 1.

**TABLE 1.** Recipient demographics based on 2 time groups

Variable	Overall	NT (12 pm–12 am) (N = 16 455)	DT (12 am–12 pm) (N = 15 258)	*P*
Age, y	56 (47–63)	56 (46–63)	56 (47–63)	0.106
Male sex	23 469 (74%)	12 139 (73.8%)	11 330 (74.3%)	0.331
Race				0.018
White	20 908 (65.9%)	10 828 (65.8%)	10 080 (66.1%)	
Black	6756 (21.3%)	3448 (21%)	3308 (21.7%)	
Other	4049 (12.8%)	2179 (13.2%)	1870 (12.3%)	
BMI, kg/m^2^	27.2 (23.8–30.9)	27.2 (23.8–30.9)	27.2 (23.8–30.9)	0.956
Weight	82.5 (70.3–95.3)	82.4 (70.3–95.3)	82.6 (70.6–95.3)	0.703
Former smoker >20 pack years	14 729 (46.6%)	7582 (46.2%)	7147 (47%)	0.18
Diabetes	8738 (27.6%)	4552 (27.7%)	4186 (27.5%)	0.642
Creatinine, mg/dL	1.2 (0.9–1.4)	1.1 (0.9–1.4)	1.2 (0.9–1.5)	<0.001
GFR, mL/min/1.73 m^2^	61.4 (46.6–81.6)	62 (47.2–82.3)	60.6 (45.9–80.5)	<0.001
Preoperative dialysis	713 (2.3%)	378 (2.3%)	335 (2.2%)	0.56
Diagnosis				0.324
Ischemic dilated cardiomyopathy	11 065 (34.9%)	5747 (34.9%)	5318 (34.9%)	
Nonischemic dilated cardiomyopathy	16 508 (52.1%)	8604 (52.3%)	7904 (51.8%)	
Other	4140 (13.1%)	2104 (12.8%)	2036 (13.3%)	
Blood group				0.679
A	12 828 (40.5%)	6631 (40.3%)	6197 (40.6%)	
B	4715 (14.9%)	2476 (15%)	2239 (14.7%)	
AB	1737 (5.5%)	886 (5.4%)	851 (5.6%)	
O	12 433 (39.2%)	6462 (39.3%)	5971 (39.1%)	
IV inotropes	12 091 (38.1%)	6297 (38.3%)	5794 (38%)	0.598
Mean pulmonary artery pressure, mm Hg	26 (20–34)	26 (20–34)	26 (20–34)	0.682
Cardiac output	4.4 (3.5–5.3)	4.4 (3.5–5.3)	4.4 (3.5–5.3)	0.672
PCWP	17 (11–24)	17 (11–24)	17 (11–24)	0.731
PRA	0 (0–2)	0 (0, 2)	0 (0–2)	0.71
Days on waitlist	80 (21–250)	78 (20–249)	82 (22–252)	0.002
Preoperative mechanical support				0.308
No mechanical support	14 742 (46.5%)	7638 (46.4%)	7104 (46.6%)	
Durable LVAD	11 480 (36.2%)	5905 (35.9%)	5575 (36.5%)	
Pre-IABP	3402 (10.7%)	1796 (10.9%)	1606 (10.5%)	
Temporary VAD	483 (1.5%)	263 (1.6%)	220 (1.4%)	
BIVAD	728 (2.3%)	370 (2.2%)	358 (2.3%)	
TAH	269 (0.8%)	147 (0.9%)	122 (0.8%)	
ECMO	609 (1.9%)	336 (2%)	273 (1.8%)	

BIVAD, biventricular assist device; BMI, body mass index; DT, daytime; ECMO, extracorporeal membrane oxygenation; GFR, glomerular filtration rate; IABP, intra-aortic balloon pump; IV, intravenous; LVAD, left ventricular assist device; NT, nighttime; PCWP, pulmonary capillary wedge pressure; PRA, panel-reactive antibody; TAH, total artificial heart; VAD, ventricular assist device.

There was no difference in baseline comorbidities in either group. DT donors more often had trauma as their cause of death, whereas NT donors more often died from neurologic causes (seizure/cerebrovascular accident), drug overdose, asphyxiation, or cardiovascular causes. DT donors less often had a clinical infection while they were more likely to have a pulmonary infection. Additional donor characteristics can be seen in Table 2.

**TABLE 2.** Donor demographics based on 2 time groups

Variable	Overall	NT (12 pm–12 am) (N = 16 455)	DT (12 am–12 pm) (N = 15 258)	*P*
Age	30 (23–40)	31 (23–40)	30 (22–40)	0.335
Male sex	22 541 (71.1%)	11 683 (71%)	10 858 (71.2%)	0.759
Ethnicity				0.13
White	20 375 (64.2%)	10 594 (64.4%)	9781 (64.1%)	
Black	5158 (16.3%)	2720 (16.5%)	2438 (16%)	
Hispanic	5323 (16.8%)	2683 (16.3%)	2640 (17.3%)	
Asian	534 (1.7%)	286 (1.7%)	248 (1.6%)	
Other	323 (1%)	172 (1%)	151 (1%)	
CDC high risk	6938 (21.9%)	3648 (22.2%)	3290 (21.6%)	0.21
Coronary artery disease	875 (2.8%)	440 (2.7%)	435 (2.9%)	0.354
Smoking history	4100 (13.1%)	2106 (13%)	1994 (13.3%)	0.474
Recent cocaine use	6288 (20.2%)	3262 (20.2%)	3026 (20.2%)	0.998
Diabetes	1125 (3.6%)	582 (3.6%)	543 (3.6%)	0.951
Donor HgbA1C	5.3 (5–5.5)	5.3 (5–5.5)	5.3 (5–5.5)	0.302
Donor diabetes duration, y				0.054
0–5	567 (55.4%)	304 (56.8%)	263 (53.8%)	
6–10	179 (17.5%)	102 (19.1%)	77 (15.7%)	
>10	278 (27.1%)	129 (24.1%)	149 (30.5%)	
Hypertension	4809 (15.3%)	2537 (15.5%)	2272 (15%)	0.186
Body mass index	26.4 (23.3–30.4)	26.4 (23.3–30.5)	26.4 (23.4–30.4)	0.78
Donor cause of death				0.006
Neuro (seizure/CVA)	6583 (20.8%)	3483 (21.2%)	3100 (20.3%)	
Drug overdose	4111 (13%)	2198 (13.4%)	1913 (12.5%)	
Asphyxiation	1641 (5.2%)	862 (5.2%)	779 (5.1%)	
Cardiovascular	2401 (7.6%)	1272 (7.7%)	1129 (7.4%)	
Trauma (GSW/stab/blunt)	15 479 (48.8%)	7846 (47.7%)	7633 (50%)	
Drowning	189 (0.6%)	104 (0.6%)	85 (0.6%)	
Other	1309 (4.1%)	690 (4.2%)	619 (4.1%)	
Donor bloodstream infection	2827 (8.9%)	1475 (9%)	1352 (8.9%)	0.763
Donor clinical infection	21 838 (69.5%)	11 153 (68.4%)	10 685 (70.6%)	<0.001
Donor pulmonary infection	19 575 (61.7%)	9982 (60.7%)	9593 (62.9%)	<0.001
Donor creatinine	1 (0.8–1.4)	1 (0.8–1.4)	1 (0.8–1.4)	0.4
Donor alcohol use	5208 (16.8%)	2710 (16.8%)	2498 (16.7%)	0.842
Donor extracranial cancer	55 (0.2%)	28 (0.2%)	27 (0.2%)	0.992
Donor MI history	193 (0.6%)	96 (0.6%)	97 (0.6%)	0.593
Donor antihypertensive 24 h pre-XC	9665 (30.5%)	5089 (30.9%)	4576 (30%)	0.075
Donor inotropes	13 672 (43.2%)	7058 (43%)	6614 (43.4%)	0.441
Ejection fraction	60 (55–65)	60 (55–65)	60 (55–65)	0.34

CDC, Center for Disease Control; CVA, cerebrovascular accident; DT, daytime; GSW, gunshot wound; HgA1c, hemoglobin A1c; MI, myocardial infarction; NT, nighttime; XC, cross-clamp.

**FIGURE 1.**
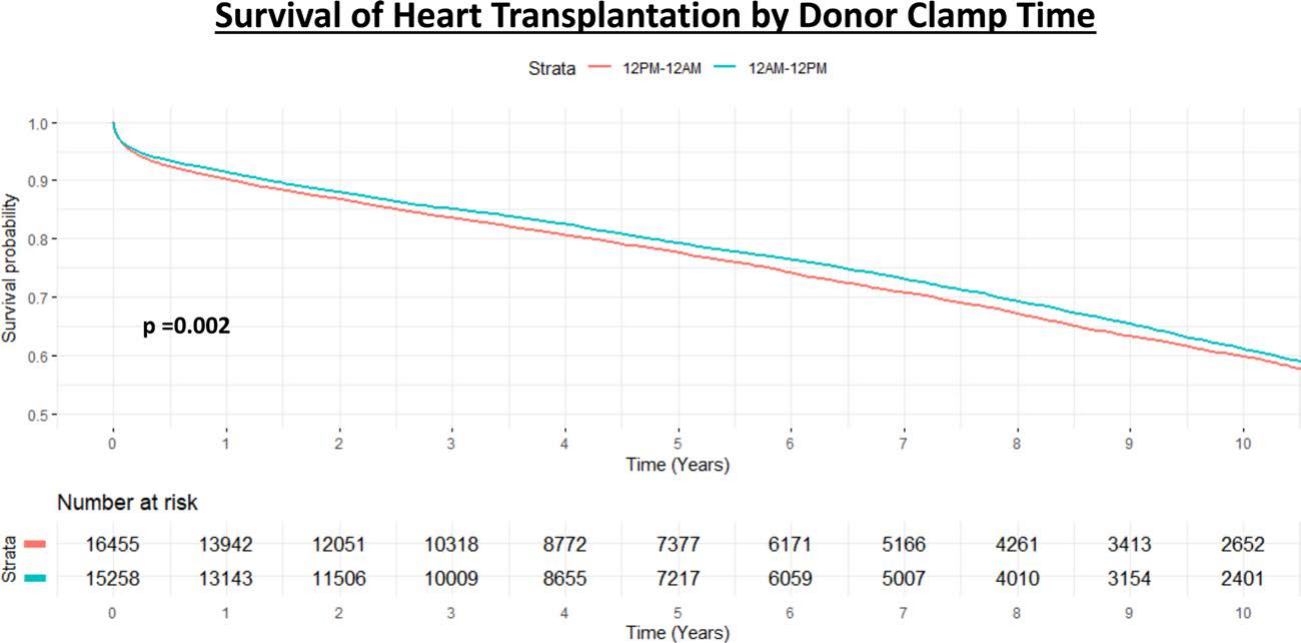
Kaplan-Meier analysis of survival after heart transplantation stratified by donor cross-clamp time (12 am–12 pm or 12 pm–12 am).

In regard to transplant characteristics, DT transplants were more likely to occur at a high-volume center, have less distance traveled, and have shorter LOS. There was no difference in acute treated rejection, rejection in the first year between groups, nor postoperative dialysis, stroke, or pacemaker. Additionally, the cause of death did not differ significantly among the groups. Additional operative outcomes and characteristics can be seen in Table 3. Unadjusted survival analysis with Kaplan-Meier methods demonstrated that patients who received hearts recovered between 12 am and 12 pm had significantly higher survival than those who received hearts recovered between 12 pm and 12 am (*P* = 0.002; Figure 1). Survival estimates for the 12 am to 12 pm group were 91.4% (95% confidence interval [CI], 91.0%-91.9%), 79.2% (95% CI, 78.5%-79.9%), and 61.1% (95% CI, 60.0%-62.2%) at 1, 5, and 10 y respectively, whereas they were 90.2% (95% CI, 89.7%-90.7%), 77.6% (95% CI, 76.9%-78.3%), and 59.2% (95% CI, 58.8%-60.9%) for the 12 pm to 12 am group.

**TABLE 3.** Operative characteristics and postoperative outcomes based on 2 time groups

Variable	Overall	NT (12 pm–12 am) (N = 16 455)	DT (12 am–12 pm) (N = 15 258)	*P*
Gender mismatch	7570 (23.9%)	3920 (23.8%)	3650 (23.9%)	0.846
Average yearly center volume	22.7 (16.1–41.9)	22.7 (15.5–41.9)	22.7 (16.1–41.9)	0.005
Distance traveled, nautical miles	114 (17–315)	118 (19–317)	111 (15–314)	0.007
Ischemia time, h	3.2 (2.5–3.9)	3.2 (2.5–3.8)	3.3 (2.5–3.9)	0.456
Length of stay, d	15 (11–23)	15 (11–23)	15 (11–22)	0.002
In-hospital mortality	1396 (4.4%)	765 (4.7%)	631 (4.2%)	0.028
Postoperative dialysis	3522 (11.2%)	1843 (11.2%)	1679 (11.1%)	0.647
Postoperative stroke	897 (2.8%)	460 (2.8%)	437 (2.9%)	0.71
Postoperative pacemaker	911 (2.9%)	481 (2.9%)	430 (2.8%)	0.593
Acute rejection before discharge				0.279
Yes and treated with immunosuppressant	3394 (10.7%)	1748 (10.6%)	1646 (10.8%)	
Yes and not treated with immunosuppressant	2592 (8.2%)	1309 (8%)	1283 (8.4%)	
No	25 723 (81.1%)	13 395 (81.4%)	12 328 (80.8%)	
Acute rejection before discharge (simple)	25 723 (81.1%)	13 395 (81.4%)	12 328 (80.8%)	0.165
Treated for rejection in first year	5388 (19.5%)	2786 (19.6%)	2602 (19.4%)	0.644
Cause of death				0.506
Cardio/cerebrovascular	1908 (25.2%)	1022 (25.1%)	886 (25.3%)	
Graft failure: acute rejection	324 (4.3%)	190 (4.7%)	134 (3.8%)	
Graft failure: chronic rejection	211 (2.8%)	107 (2.6%)	104 (3%)	
Graft failure: other	118 (1.6%)	58 (1.4%)	60 (1.7%)	
Graft failure: primary	333 (4.4%)	188 (4.6%)	145 (4.1%)	
Infection	1447 (19.1%)	775 (19.1%)	672 (19.2%)	
Malignancy	866 (11.4%)	471 (11.6%)	395 (11.3%)	
Other	1803 (23.8%)	970 (23.8%)	833 (23.8%)	
Pulmonary	558 (7.4%)	287 (7.1%)	271 (7.7%)	

DT, daytime; NT, nighttime.

**FIGURE 2.**
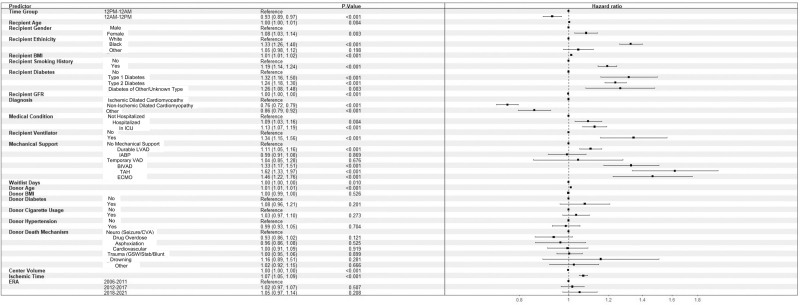
Forest plot demonstrating hazard ratio after heart transplantation with 2 donor cross-clamp time groups. BIVAD, biventricular assist device; BMI, body mass index; CVA, cerebrovascular accident; ECMO, extracorporeal membrane oxygenation; GFR, glomerular filtration rate; GSW, gunshot wound; IABP, intra-aortic balloon pump; ICU, intensive care unit; LVAD, left ventricular assist device; TAH, total artificial heart; VAD, ventricular assist device.

Following adjustment, the time of 12 am to 12 pm was independently associated with higher survival than those who received hearts recovered between 12 pm and 12 am (hazard ratio [HR]: 0.93; 95% CI, 0.89-0.97; *P* < 0.001). For recipients, variables that were significantly associated with decreased long-term survival included age, female sex, Black ethnicity, BMI, smoking history, diabetes, and ventilator use. Additionally, hospitalization or status compared with nonhospitalization was significantly associated with decreased long-term survival. In terms of MCS before transplantation, durable LVAD, BIVAD, TAH, and ECMO were all significantly associated with decreased long-term survival. Finally, donor age and ischemic time were also associated with decreased survival. A recipient diagnosis other than ischemic cardiomyopathy was significantly associated with increased long-term survival. Additional results can be seen in Figure 2.

### Results Based on the 5 Category Time Split

Regarding heart transplant recipients, there were 5302 (16.72%) patients in T1, 3498 (11.03%) in T2, 7657 (24.14%) in T3, 6897 (21.75%) in T4, and 8359 (26.36%) in T5. T2 recipients had significantly higher BMI, whereas recipients in T4 had significantly lower creatinine and higher GFR. Recipients in T2 spent the longest days on the waitlist, had the highest incidence of pretransplant LVAD, had the lowest incidence of pretransplant intra-aortic balloon pump, and had the highest incidence of pretransplant ECMO use. Additional demographics can be seen in Table 4.

**TABLE 4.** Recipient demographics based on 5 time groups

Variable	Overall (N = 31 713)	T1 (4 am–8 am) (N = 5302)	T2 (8 am–11 am) (N = 3498)	T3 (11 am–5 pm) (N = 7657)	T4 (5 pm–10 pm) (N = 6897)	T5 (10 pm–4 am) (N = 8359)	P
Age, y	56 (47–63)	56 (47–63)	56 (46–63)	56 (46–63)	56 (46–63)	56 (47–63)	0.439
Male sex	23 469 (74%)	3968 (74.8%)	2575 (73.6%)	5616 (73.3%)	5061 (73.4%)	6249 (74.8%)	0.099
Race							0.42
White	20 908 (65.9%)	3553 (67%)	20 (66%)	4993 (65.2%)	42 (66.1%)	5490 (65.7%)	
Black	6756 (21.3%)	1098 (20.7%)	744 (21.3%)	1685 (22%)	1424 (20.6%)	1805 (21.6%)	
Other	4049 (12.8%)	651 (12.3%)	444 (12.7%)	979 (12.8%)	911 (13.2%)	1064 (12.7%)	
BMI, kg/m^2^	27.2 (23.8–30.9)	27.1 (23.8–30.7)	27.4 (24–31.2)	27.3 (23.9–31)	27.1 (23.8–30.9)	27.1 (23.8–30.7)	0.026
Weight	82.5 (70.3–95.3)	82.5 (70.4–95.3)	83.3 (70.8–95.4)	82.1 (70.4–95.3)	82.2 (70.3–95.3)	82.3 (70.4–95.2)	0.434
Former smoker > 20 pack years	14 729 (46.6%)	2466 (46.7%)	1661 (47.6%)	3511 (46%)	3165 (46%)	3926 (47.1%)	0.367
Diabetes	8738 (27.6%)	1437 (27.1%)	944 (27%)	2141 (28%)	1887 (27.4%)	2329 (27.9%)	0.667
Creatinine, mg/dL	1.2 (0.9–1.4)	1.2 (0.9–1.5)	1.2 (0.9–1.4)	1.2 (0.9–1.4)	1.1 (0.9–1.4)	1.2 (1–1.5)	<0.001
GFR, mL/min/1.73 m^2^	61.4 (46.6–81.6)	60.3 (45.9–80)	61.2 (46.2–81.7)	61.9 (46.7–82.3)	62.7 (47.5–82.9)	60.6 (46.2–79.9)	<0.001
Preoperative dialysis	713 (2.3%)	105 (2%)	75 (2.1%)	180 (2.4%)	150 (2.2%)	203 (2.4%)	0.447
Diagnosis							0.247
Ischemic dilated cardiomyopathy	11 065 (34.9%)	1856 (35%)	1191 (34%)	2609 (34.1%)	2416 (35%)	2993 (35.8%)	
Nonischemic dilated cardiomyopathy	16 508 (52.1%)	2724 (51.4%)	1840 (52.6%)	4027 (52.6%)	3587 (52%)	4330 (51.8%)	
Other	4140 (13.1%)	722 (13.6%)	467 (13.4%)	1021 (13.3%)	894 (13%)	1036 (12.4%)	
Blood group							0.778
A	12 828 (40.5%)	2172 (41%)	1413 (40.4%)	3071 (40.1%)	2812 (40.8%)	3360 (40.2%)	
B	4715 (14.9%)	779 (14.7%)	519 (14.8%)	1156 (15.1%)	1046 (15.2%)	1215 (14.5%)	
AB	1737 (5.5%)	313 (5.9%)	183 (5.2%)	405 (5.3%)	360 (5.2%)	476 (5.7%)	
O	12 433 (39.2%)	2038 (38.4%)	1383 (39.5%)	3025 (39.5%)	2679 (38.8%)	3308 (39.6%)	
IV inotropes	12 091 (38.1%)	2030 (38.3%)	1265 (36.2%)	2887 (37.7%)	2612 (37.9%)	3297 (39.4%)	0.013
Mean pulmonary artery pressure, mm Hg	26 (20–34)	26.7 (20–34)	26 (20–34)	26 (20–34)	27 (20–34)	27 (20–34)	0.01
Cardiac output	4.4 (3.5–5.3)	4.3 (3.5–5.3)	4.4 (3.6–5.4)	4.4 (3.5–5.3)	4.4 (3.5–5.3)	4.3 (3.5–5.3)	0.008

BMI, body mass index; GFR, glomerular filtration rate; IV, intravenous.

Donors in T2 were more often White and were considered high risk by the Center for Disease Control. No significant differences were observed in donor incidence of smoking, recent cocaine use, diabetes, and coronary artery disease. However, donors in T2 had the highest incidence of diabetes lasting >10 y. Donors in T4 had the highest incidence of death by neurologic causes, whereas donors in T5 had the highest incidence of death by cardiovascular causes. Donors in T1 had the highest incidence of both diagnosed clinical infection and pulmonary infection. Additional characteristics can be seen in Table 5.

**TABLE 5.** Donor demographics based on 5 time groups

Variable	Overall (N = 31 713)	T1 (4 am–8 am) (N = 5302)	T2 (8 am–11 am) (N = 3498)	T3 (11 am–5 pm) (N = 7657)	T4 (5 pm–10 pm) (n = 6897)	T5 (10 pm–4 am) (N = 8359)	*P*
Age	30 (23–40)	31 (23–41)	30 (23–40)	30 (22–40)	30 (23–40)	30 (22–41)	0.401
Male sex	22 541 (71.1%)	3791 (71.5%)	2510 (71.8%)	5434 (71%)	4878 (70.7%)	5928 (70.9%)	0.771
Ethnicity							<0.001
White	20 375 (64.2%)	3376 (63.7%)	2292 (65.5%)	5010 (65.4%)	4462 (64.7%)	5235 (62.6%)	
Black	5158 (16.3%)	859 (16.2%)	605 (17.3%)	1260 (16.5%)	1109 (16.1%)	1325 (15.9%)	
Hispanic	5323 (16.8%)	920 (17.4%)	527 (15.1%)	1205 (15.7%)	1111 (16.1%)	1560 (18.7%)	
Asian	534 (1.7%)	90 (1.7%)	41 (1.2%)	118 (1.5%)	137 (2%)	148 (1.8%)	
Other	323 (1%)	57 (1.1%)	33 (0.9%)	64 (0.8%)	78 (1.1%)	91 (1.1%)	
CDC high risk	6938 (21.9%)	1131 (21.4%)	811 (23.2%)	1761 (23%)	1499 (21.7%)	1736 (20.8%)	0.003
Coronary artery disease	875 (2.8%)	145 (2.7%)	112 (3.2%)	213 (2.8%)	175 (2.5%)	230 (2.8%)	0.427
Smoking history	4100 (13.1%)	729 (13.9%)	431 (12.5%)	960 (12.7%)	887 (13%)	1093 (13.2%)	0.242
Recent cocaine use	6288 (20.2%)	1046 (20.1%)	717 (20.9%)	1499 (19.9%)	1406 (20.8%)	1620 (19.7%)	0.414
Diabetes	1125 (3.6%)	197 (3.7%)	123 (3.5%)	255 (3.3%)	257 (3.7%)	293 (3.5%)	0.699
Donor HgbA1c	5.3 (5–5.5)	5.3 (5.1–5.6)	5.3 (5–5.5)	5.3 (5–5.5)	5.3 (5–5.5)	5.3 (5–5.5)	0.426
Donor diabetes duration, y							0.048
0–5	567 (55.4%)	101 (57.1%)	48 (42.9%)	132 (56.4%)	131 (56.5%)	155 (57.6%)	
6–10	179 (17.5%)	21 (11.9%)	25 (22.3%)	49 (20.9%)	38 (16.4%)	46 (17.1%)	
>10	278 (27.1%)	55 (31.1%)	39 (34.8%)	53 (22.6%)	63 (27.2%)	68 (25.3%)	
Hypertension	4809 (15.3%)	813 (15.4%)	507 (14.6%)	1136 (14.9%)	1085 (15.8%)	1268 (15.3%)	0.455
Body mass index	26.4 (23.3–30.4)	26.5 (23.3–30.6)	26.4 (23.4–30.4)	26.4 (23.3–30.4)	26.4 (23.3–30.4)	26.4 (23.3–30.4)	0.945
Donor cause of death							<0.001
Neuro (seizure/CVA)	6583 (20.8%)	1082 (20.4%)	677 (19.4%)	1542 (20.1%)	1494 (21.7%)	1788 (21.4%)	
Drug overdose	4111 (13%)	613 (11.6%)	493 (14.1%)	1058 (13.8%)	916 (13.3%)	1031 (12.3%)	
Asphyxiation	1641 (5.2%)	299 (5.6%)	146 (4.2%)	435 (5.7%)	338 (4.9%)	423 (5.1%)	
Cardiovascular	2401 (7.6%)	387 (7.3%)	271 (7.7%)	580 (7.6%)	515 (7.5%)	648 (7.8%)	
Trauma (GSW/stab/blunt)	15 479 (48.8%)	2688 (50.7%)	1751 (50.1%)	3639 (47.5%)	3287 (47.7%)	4114 (49.2%)	
Drowning	189 (0.6%)	25 (0.5%)	20 (0.6%)	40 (0.5%)	49 (0.7%)	55 (0.7%)	
Other	1309 (4.1%)	208 (3.9%)	140 (4%)	363 (4.7%)	298 (4.3%)	300 (3.6%)	
Donor bloodstream infection	2827 (8.9%)	460 (8.7%)	318 (9.1%)	714 (9.3%)	603 (8.7%)	732 (8.8%)	0.627
Donor clinical infection	21 838 (69.5%)	3717 (70.7%)	2449 (70.7%)	5235 (68.9%)	4671 (68.3%)	5766 (69.6%)	0.021
Donor pulmonary infection	19 575 (61.7%)	3342 (63%)	2197 (62.8%)	4683 (61.2%)	4175 (60.5%)	5178 (61.9%)	0.027
Donor creatinine	1 (0.8–1.4)	1 (0.8–1.4)	1 (0.8–1.4)	1 (0.8–1.4)	1 (0.8–1.4)	1 (0.8–1.4)	0.378
Donor alcohol use	5208 (16.8%)	870 (16.7%)	584 (17.1%)	1260 (16.8%)	1123 (16.6%)	1371 (16.7%)	0.987
Donor extracranial cancer	55 (0.2%)	13 (0.2%)	7 (0.2%)	11 (0.1%)	10 (0.1%)	14 (0.2%)	0.65
Donor MI history	193 (0.6%)	25 (0.5%)	25 (0.7%)	41 (0.5%)	46 (0.7%)	56 (0.7%)	0.42
Donor antihypertensives 24 h pre-XC	9665 (30.5%)	1540 (29.1%)	1112 (31.8%)	2403 (31.4%)	2138 (31%)	2472 (29.6%)	0.005
Donor inotropes	13 672 (43.2%)	2237 (42.3%)	1555 (44.5%)	3287 (43%)	2931 (42.6%)	3662 (43.9%)	0.117
Ejection fraction	60 (55–65)	60 (56–65)	60 (55–65)	60 (55–65)	60 (55–65)	60 (55–65)	0.301

CDC, Center for Disease Control; CVA, cerebrovascular accident; GSW, gunshot wound; HgA1c, hemoglobin A1c; MI, myocardial infarction; XC, cross-clamp.

In regard to transplant characteristics, there was the highest incidence of gender mismatch in T5, the greatest center volume in T1, the further distance traveled in T3, and the shortest ischemic time in T2. In-hospital mortality, postoperative dialysis, stroke, pacemaker, acute rejection before discharge, treated rejection in the first year, and cause of death did not differ significantly between groups. Additional operative characteristics and outcomes can be seen in Table 6.

**TABLE 6.** Operative characteristics and postoperative outcomes based on 5 time groups

Variable	Overall (N = 31 713)	T1 (4 am–8 am) (N = 5302)	T2 (8 am–11 am) (N = 3498)	T3 (11 am–5 pm) (N = 7657)	T4 (5 pm–10 pm) (N = 6897)	T5 (10 pm–4 am) (N = 8359)	*P*
Gender mismatch	7570 (23.9%)	1295 (24.4%)	749 (21.4%)	1770 (23.1%)	1651 (23.9%)	2105 (25.2%)	<0.001
Average yearly center volume	22.7 (16.1–41.9)	22.8 (16.5–43.4)	22.7 (16.1–35.8)	22.7 (15.5–37.3)	22.7 (15.5–41.9)	22.7 (16.1–42.6)	<0.001
Distance traveled, nautical miles	114 (17–315)	115 (16–318)	101 (10–299)	130 (19–326)	112 (19–315)	111 (19–310)	<0.001
Ischemia time, h	3.2 (2.5–3.9)	3.3 (2.5–3.9)	3.1 (2.3–3.8)	3.3 (2.5–3.9)	3.2 (2.5–3.8)	3.3 (2.5–3.9)	<0.001
Length of stay, d	15 (11–23)	15 (10–22)	15 (11–23)	15 (11–23)	15 (11–23)	15 (10–23)	<0.001
In-hospital mortality	1396 (4.4%)	217 (4.1%)	152 (4.4%)	351 (4.6%)	320 (4.7%)	356 (4.3%)	0.523
Postoperative dialysis	3522 (11.2%)	546 (10.3%)	393 (11.4%)	845 (11.1%)	805 (11.7%)	933 (11.2%)	0.201
Postoperative stroke	897 (2.8%)	168 (3.2%)	88 (2.5%)	215 (2.8%)	201 (2.9%)	225 (2.7%)	0.384
Postoperative pacemaker	911 (2.9%)	151 (2.9%)	87 (2.5%)	217 (2.8%)	205 (3%)	251 (3%)	0.611
Acute rejection before discharge							0.183
Yes and treated with immunosuppressant	3394 (10.7%)	584 (11%)	369 (10.5%)	844 (11%)	704 (10.2%)	893 (10.7%)	
Yes and not treated with immunosuppressant	2592 (8.2%)	451 (8.5%)	308 (8.8%)	628 (8.2%)	578 (8.4%)	627 (7.5%)	
No	25 723 (81.1%)	4267 (80.5%)	2821 (80.6%)	6183 (80.8%)	5614 (81.4%)	6838 (81.8%)	
Acute rejection before discharge (simple)	5986 (18.9%)	1035 (19.5%)	677 (19.4%)	1472 (19.2%)	1282 (18.6%)	1520 (18.2%)	0.235
Treated for rejection in first year	,388 (19.5%)	912 (19.5%)	581 (18.9%)	1287 (19.4%)	1146 (19.3%)	1462 (20.1%)	0.663
Cause of death							0.523
Cardio/cerebrovascular	1908 (25.2%)	305 (26.2%)	181 (23.1%)	442 (24.7%)	455 (26.3%)	525 (25%)	
Graft failure: acute rejection	324 (4.3%)	37 (3.2%)	31 (4%)	83 (4.6%)	84 (4.8%)	89 (4.2%)	
Graft failure: chronic rejection	211 (2.8%)	34 (2.9%)	19 (2.4%)	47 (2.6%)	48 (2.8%)	63 (3%)	
Graft failure: other	118 (1.6%)	23 (2%)	9 (1.1%)	34 (1.9%)	22 (1.3%)	30 (1.4%)	
Graft failure: primary	333 (4.4%)	47 (4%)	44 (5.6%)	83 (4.6%)	80 (4.6%)	79 (3.8%)	
Infection	1447 (19.1%)	235 (20.2%)	167 (21.3%)	321 (17.9%)	313 (18.1%)	411 (19.6%)	
Malignancy	866 (11.4%)	125 (10.7%)	87 (11.1%)	205 (11.4%)	195 (11.3%)	254 (12.1%)	
Other	1803 (23.8%)	275 (23.6%)	178 (22.7%)	436 (24.3%)	424 (24.5%)	490 (23.4%)	
Pulmonary	558 (7.4%)	83 (7.1%)	67 (8.6%)	140 (7.8%)	112 (6.5%)	156 (7.4%)	

**FIGURE 3.**
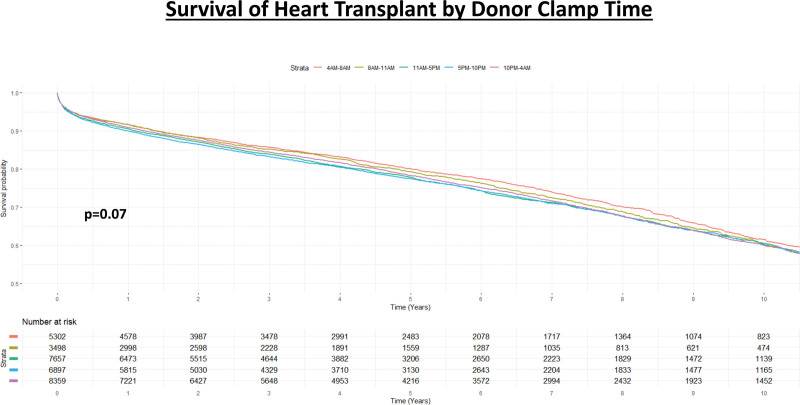
Kaplan-Meier analysis of survival after heart transplantation stratified by donor cross-clamp time (4 am–8 am; 8 am–11 am; 11 am–5 pm; 5 pm–10 pm; 10 pm–4 am).

**FIGURE 4.**
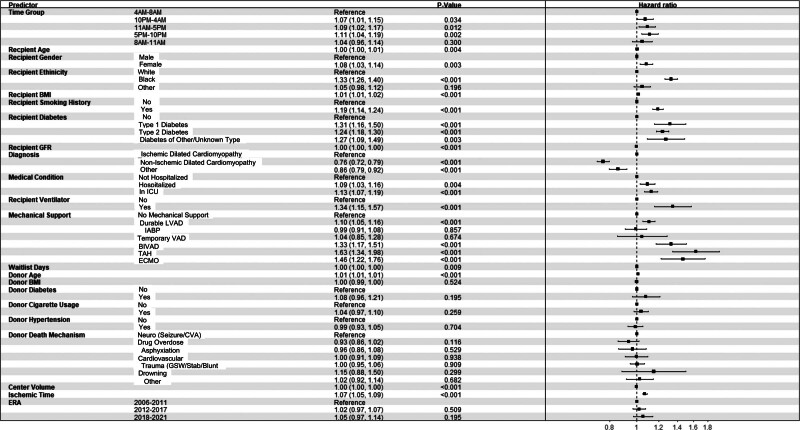
Forest plot demonstrating hazard ratio after heart transplantation with 5 donor cross-clamp time groups. BIVAD, biventricular assist device; BMI, body mass index; CVA, cerebrovascular accident; ECMO, extracorporeal membrane oxygenation; GFR, glomerular filtration rate; GSW, gunshot wound; IABP, intra-aortic balloon pump; ICU, intensive care unit; LVAD, left ventricular assist device; TAH, total artificial heart; VAD, ventricular assist device.

Unadjusted survival analysis with Kaplan-Meier methods demonstrated that there was no significant difference in long-term survival among the 5 groups (*P* = 0.07; Figure 3). However, after adjustment, the groups T3 (HR: 1.09; 95% CI, 1.02-1.17; *P* = 0.012), T4 (HR: 1.11; 95% CI, 1.04-1.19; *P* = 0.002), and T5 (HR: 1.07; 95% CI, 1.01-1.15; *P* = 0.034) were all independently associated with decreased long-term survival compared with T1. Variables that were significantly associated with decreased long-term survival included increased recipient age, female recipient, Black race, BMI, smoking history, diabetes, postoperative ventilator use, and ischemic time. The use of MCS posttransplant, specifically durable LVAD, BIVAD, TAH, and ECMO carried a significant risk of increased long-term mortality. Variables associated with increased long-term survival were recipient GFR, a diagnosis of nonischemic dilated cardiomyopathy compared with ischemic cardiomyopathy, and yearly center volume. Additional results can be seen in Figure 4.

## DISCUSSION

Among a variety of donor cross-clamps, several have statistical significance in terms of decreasing or increasing long-term survival. We found that patients who received hearts recovered between 12 am and 12 pm had significantly higher survival than those who received hearts recovered between 12 pm and 12 am. Further analysis revealed that when donor clamp time occurred outside the hours of 4 am to 11 am, there was an associated decrease in long-term survival; however, it is to note that all HRs were relatively small. Death due to acute rejection was higher in the 12 pm to 12 am group, whereas other causes of death, including cardiocerebrovascular, chronic rejection, primary graft failure, infection, and pulmonary causes, were insignificant indicating a possible mechanism because of early donor immune-related factor. Although the statistical significance only resulted in a 1% to 2% change in survival over several years, this small change could still have an impact on the thousands of heart transplantation recipients every year, calling to attention a need for further research to determine underlying molecular mechanisms in preclinical studies.

Current biologic evidence indicate circadian activators and repressors govern organ functional oscillation in mammals. In brief, activators were apparent during the middle of the day with occupancy of CLOCK–BMAL1, whereas on the contrary, repressors PER1, PER2, and CRY2 bind genome-wide between midnight to sunrise.^4^ Together, both positive and negative regulators form a cell-autonomous canonical feedback loop. Although cardiometabolic functional oscillations were previously recognized,^6^ whether circadian activators modulate cardiac-related immunity is mostly unexplored. There has been a recent focus on determining whether circadian rhythms (or transplantation timing) affect heart transplantation outcomes. Previously, George et al^1^ found no difference in DT or NT heart transplantation in 30-d, 90-d, and 1-y survival.^1^ Their work was further substantiated in single-institutional data, where heart transplantation outcomes were analyzed on the basis of normal or outside working hours. Nishida et al^2^ found no significant differences in 1- or 3-y survival, and furthermore, they found no difference in postoperative stroke, graft dysfunction requiring ECMO use, or dialysis use. Finally, Immohr et al^3^ found no difference in morning, afternoon, and NT transplantation in terms of 30-d and 1-y survival. Interestingly, however, they did show that reexploration for bleeding was slightly higher in the afternoon cohort.^3^ In contradiction to previous work, we found that donor cross-clamp times between 12 pm and 12 am was associated with increased mortality, in addition to times outside of 4 am to 11 am. Furthermore, those transplanted outside the hours of 4 am to 11 am had significantly longer distances traveled, ischemic times, gender mismatch, and LOS while also trending to have higher in-hospital mortality, postoperative dialysis, and pacemaker placement.

This analysis identified that an optimal procurement time could possibly be between 4 am and 8 am. Although this may be an optimal time to procure heart allografts, there are other factors to consider. First, the recipient; second, the donor; and finally, the members of the transplant team. Although the first 2 could possibly be controlled, the latter is a confounder is difficult to address and warrants further study. Transplant surgeons are often on call 4 d/wk and can work upward of 70 h/wk.^7,8^ Furthermore, transplant surgeons sleep significantly less than the average American, averaging 6.33 h a night.^9^ This optimal procurement time could possibly further contribute to lack of sleep among these individuals and lead to higher rates of burnout^10-15^ as well as medical errors.^16^ However, this scheduled procurement timing could possibly alleviate lack of transparent schedules among providers. Additionally, with the advent of ex vivo cardiac perfusion and its ability to mitigate the effects of ischemic time, this optimal procurement timing might only affect select members of the procurement team and not necessarily the implant team. It will be further necessary to study circadian rhythms in surgeons and providers to better understand the complex interplay of these circadian clocks and performance.

Although circadian rhythms offer a possible explanation for this finding, there are several others. This small 1% to 2% difference in survival may not be clinically significant and may represent a cofounding of a litany of factors that are difficult to elucidate in a large database. Although in general, transplantation often occurs safely at any hour of the day due inherent to the nature of transplantation centers and transplantation surgery, this observed difference could possibly represent a system-based problem within transplantation that may be remedied with adequate attention paid to often overworked staff and surgeons. Finally, as mentioned previously, the growth of ex vivo perfusion could possibly make DT surgeries possible and suspend circadian rhythms altogether, eliminating the need for optimal procurement time.^17^

### Limitations

This study has inherent limitations that affect any large database, including lack of granular data, its retrospective nature, and its subject to information and selection bias. Data regarding the treatment of acute rejection, as well as other perioperative complications, and the long-term impact of morbidity, such as dialysis and rejection, were not available, thus limiting inferences regarding quality of life. Furthermore, data regarding chronic lung allograft dysfunction were not available, preventing examination of the impact of procurement timing on chronic lung allograft dysfunction. Additionally, it is worth noting that some of the transplants were presumably performed between donors and recipients in different time zones across the country, and we were unable to account for this effect within this analysis. Finally, although circadian rhythms in brain death have not been explicitly studied, there has been an investigation into circadian rhythms in brain-injured patients. Patients who are critically ill in the intensive care unit have been shown to have no rhythmic expression,^18^ and those with brain injuries within the intensive care unit are shown to lose rhythmic expression after 1 wk.^19^ Although these studies were not in brain-dead donors, it is difficult to imagine their circadian rhythms are not disrupted in some manner, and thus, further studies are needed to determine baseline circadian function in brain dead donors.

## CONCLUSIONS

Given the independent association of donor timing and survival, after adjustment in a large national cohort, further study is warranted to elucidate the underlying reasons behind this change. Although this finding may not be clinically significant or based on system-level factors that must be remedied through administrative actions, the possibility of circadian rhythms serving a role should be further investigated. Based on circadian rhythms, we hypothesize that there may be a change in the regulation of certain critical pathways that may influence the donor or recipients’ immunologic response to transplantation and the presence of antigens that may play a role in rejection. Thus, understanding the underlying mechanisms of this observation could potentially lead to the development of effective treatments and donor procurement processes that prepare the organs under a better condition for transplantation.
